# Fracture Risk and Adjuvant Therapies in Young Breast Cancer Patients: A Population-Based Study

**DOI:** 10.1371/journal.pone.0130725

**Published:** 2015-06-24

**Authors:** Chun-Hung Chang, Shaw-Ji Chen, Chieh-Yu Liu

**Affiliations:** 1 China Medical University Hospital, Taichung, Taiwan, R.O.C; 2 Institute of Clinical Medicine, China Medical University, Taichung, Taiwan, R.O.C; 3 Department of Psychiatry, Mackay Memorial Hospital Taitung Branch, Taitung, R.O.C; 4 Institute of Medical Sciences, Tzu Chi university, Hualien, Taiwan, R.O.C; 5 Biostatistical Consulting Lab, Institute of Nursing-Midwifery, National Taipei University of Nursing and Health Sciences, Taipei, Taiwan, R.O.C; University of North Carolina School of Medicine, UNITED STATES

## Abstract

**Background:**

Breast cancer survivors have an increased risk of bone fracture. But the risk among young patients with adjuvant therapies remains unknown. This population-based study is aimed to assess the incidence and risk of fracture among young (age of 20 to 39 years) breast cancer patients who received adjuvant therapies.

**Methods:**

From January 2001 to December 2007, 5,146 newly diagnosed breast cancer patients were enrolled from the National Health Insurance Research Database (NHIRD) in Taiwan. Patients were observed for a maximum of 6 years to determine the incidence of newly onset fracture. Kaplan Meier and Cox regression analyses were used to evaluate the risk of fracture in young breast cancer patients who received adjuvant treatments.

**Results:**

Of the total 5,146 young (age of 20 to 39 years) breast cancer patients, the Cox multivariate proportional hazards analysis showed that AIs, radiotherapy, and monoclonal antibodies were significantly associated with a high risk of fracture. Moreover, patients who received AIs for more than 180 days had a high hazard ratio (HR) of 1.77 (95% CI = 0.68–4.57), and patients who received more than four radiotherapy visits had a high HR of 2.54 (95% CI = 1.07–6.06). Under the site-specific analysis, young breast cancer patients who received AIs had the highest risk of hip fracture (HR = 8.520, 95% CI = 1.711–42.432, p < 0.04), whereas patients who received radiotherapy had the highest risk of vertebral fracture (HR = 5.512, 95% CI = 1.847–16.451, p < 0.01).

**Conclusion:**

Young breast cancer patients who are receiving AIs, radiotherapy or monoclonal antibody need to be more careful for preventing fracture events. Breast cancer treatment plans are suggested to incorporate fracture prevention interventions.

## Introduction

Breast cancer is the most prevalent malignancy and the leading cause of cancer-related mortality in women worldwide [1, 2]. In Taiwan, the 5-year survival rates increased from 69.79% in 1986 to 82.85% in 2003 [33] because of early screening [4], surgery, and adjuvant therapies such as the use of selective estrogen receptor modulators (e.g. tamoxifen) [5], third-generation aromatase inhibitors (AIs; e.g. anastrozole, letrozole, or exemestane), monoclonal antibody (e.g. trastuzumab) [2, 6], chemotherapy [7], and radiotherapy [8].

An increased risk of fracture has been observed in breast cancer survivors [9–11]. However, the risk of fracture following adjuvant therapies, which are increasingly used for breast cancer treatment, has not been investigated thoroughly. Two studies have associated AIs with an increased risk of fracture in postmenopausal breast cancer patients [12, 13]; however, they did not address the risk of fracture in young patients. Conversely, tamoxifen, a selective estrogen receptor modulator, was reported to preserve the bone mineral density of the lumbar spine in postmenopausal women [14]; however, evidence on fractures has been has been conflicting [15–18]. Moreover, the risk of fracture in young breast cancer survivors receiving monoclonal antibodies, chemotherapy, and radiotherapy has not been evaluated.

Young women with breast cancer are considered a special group of breast cancer patient because they have poor prognosis, and the survival rates for women aged<40 years of age are comparatively lower than those for older women [19, 20]. Approximately 7% of women with breast cancer are diagnosed before the age of 40 years [21], but the incidence of young breast cancer increase [2]. Moreover, one study reported younger age to be an independent predictor of adverse outcomes of adjuvant therapies [21].

We investigated the risk of fracture resulting from adjuvant therapies in young breast cancer patients aged 20–39 years by retrieving claims data from the population-based retrospective database of the National Health Insurance Research Database (NHIRD) in Taiwan.

## Methods

### Database

We used available claims data from Taiwan’s National Health Insurance (NHI) program, which was launched by the Taiwan government in 1995 and provided comprehensive health care for 98.29% of its residents in 2006 [22]. The NHIRD contains comprehensive information including outpatient, inpatient, prescription drugs, and traditional Chinese medicine services. The diagnostic and procedure codes are based on the International Classification of Diseases, Ninth revision, Clinical Modification (ICD-9-CM) and Procedure Coding System (ICD-9-PCS).

### Ethics statement

The Institutional Review Board of China Medical University Hospital approved this study (CMUH103-REC3-077). The National Health Research Institutes encrypt the personal information of patients for privacy protection. The NHI Bureau and Institutional Review Board of China Medical University Hospital guarantee the confidentiality of the personal and health information of patients.

### Study population

**Fig 1:**
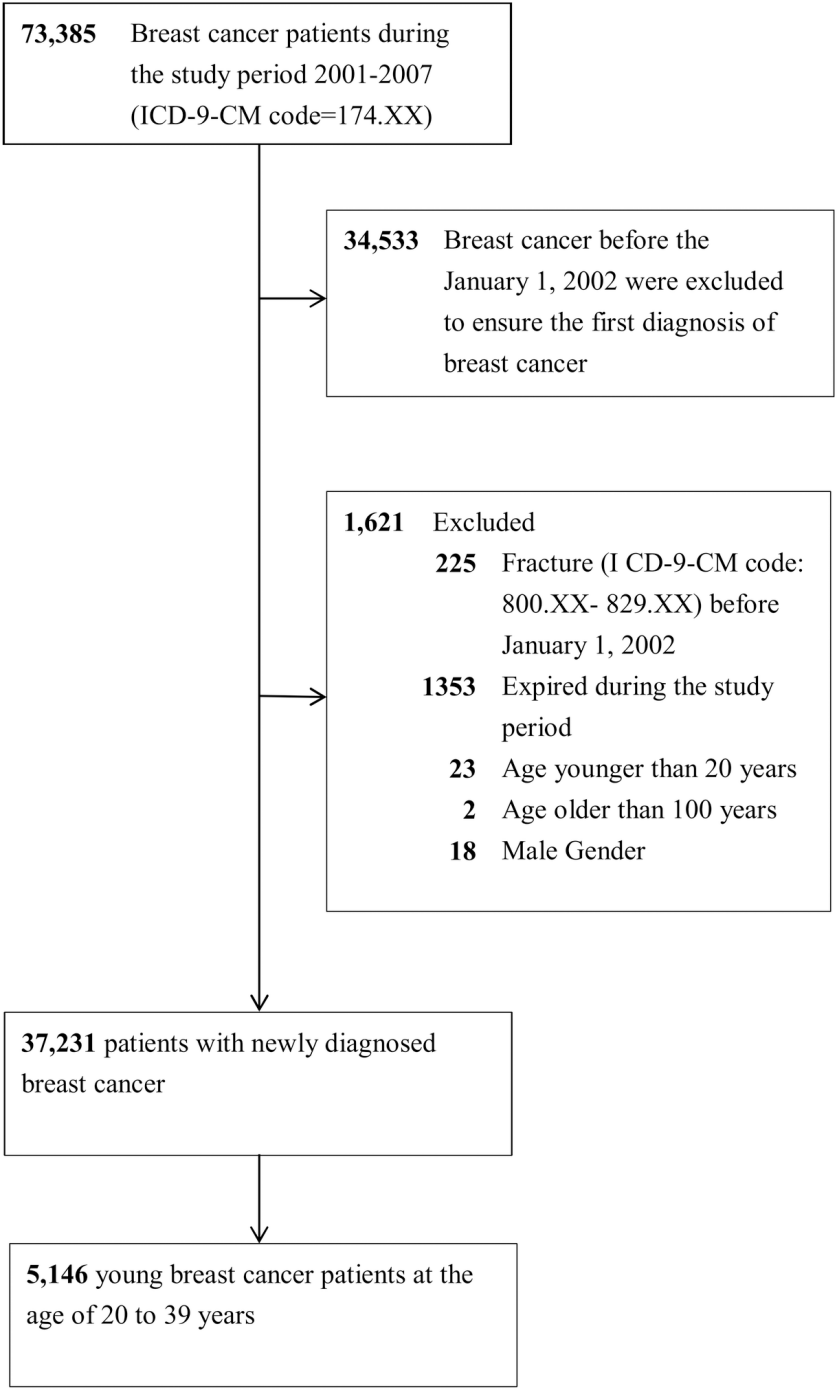
Selection of study patients.

We identified patients aged 20 to 39 years with an initial diagnosis of breast cancer (ICD-9-CM code 174.XX) between January 1, 2002 and December 31, 2007 from the NHIRD. This breast cancer cohort was followed until the date of fracture (ICD-9-CM codes 800–829), death, withdrawal from the National Health Insurance program, or the end of 2007. We further investigated the risk of fracture at three sites: hip (ICD-9-CM 820), vertebrae (ICD-9-CM 806.20–806.9), and forearm (ICD-9-CM 813) [10] (Fig 1).

### Covariate assessment

We considered adjuvant therapies including selective estrogen receptor modulator (tamoxifen), AIs (anastrozole, letrozole, and exemestane), monoclonal antibody (trastuzumab), chemotherapy, and radiotherapy according to diagnostic and procedure codes. Based on the medication (tamoxifen, anastrozole, letrozole, exemestane, and trastuzumab) prescribed to the patients, the ‘prescription duration’ (sum of prescription’s days of supply) for each patient was calculated for dosage effect evaluation. Patients who received chemotherapy (ICD-9 codes V581 and 992.5), or radiotherapy (ICD-9 codes 922 and V580) were identified. Furthermore, we defined distant metastases as metastases to the lungs, liver, brain, bones, and other organs (ICD-9 codes 197.x, 198.0, 198.1, and 198.3–198.7). The Charlson comorbidity index (CCI) was calculated to represent degree of comorbidity [23].

### Statistical analysis

The incidence densities and hazard ratio (HR) of fractures in young breast cancer patients with and without specific adjuvant treatment were calculated. Univariate and multivariate Cox proportional hazard regression were used to evaluate the effects of adjuvant therapies on the risk of fracture; the results were expressed using HRs with 95% confidence intervals (CIs). The multivariate model was used to control for age and comorbidities. We estimated the cohort-specific cumulative incidences by 1- (Kaplan Meier survival rate) for unadjusted curves and tested the differences by using log-rank tests. My Structured Query Language (MySQL) was used for extraction, linkage, and processing of the data. All statistical analyses were performed using IBM SPSS statistical software Version 20 (IBM Corp., New York, NY, USA). A two-tailed p < 0.05 was considered statistically significant.

## Results

### Clinical characteristics of the study population

Based on the selection criteria (Fig 1), this study comprised 5,146 young breast cancer patients aged 20 to 39 years diagnosed between January 1, 2002 and December 31, 2007. The mean age was 35.32 years (standard deviation, 3.83). The mean follow-up period in our study was 2.94 ± 1.69 years. The most common adjuvant treatment among breast cancer patients was tamoxifen [63.5%], chemotherapy [52.1%], and radiotherapy [24.2%]. The most common underlying diseases were hypertension (0.4%), autoimmune diseases (0.4%), and diabetes (0.3%) (Table 1).

**Table 1:** Basic profile of young breast cancer cohort (n = 5,146).

Variable	Mean±SD/Number	(%)
Age (yrs)	35.32±3.83	
Follow-up (yrs)	2.94±1.69	
Adjuvant therapies		
Selective oestrogen receptor modulator	3267	(63.5)
AIs	395	(7.7)
Monoclonal antibody	155	(3.0)
Chemotherapy	2683	(52.1)
Radiotherapy	1244	(24.2)
Comorbidities		
Hypertension	21	(0.4)
Autoimmune diseases	20	(0.4)
DM	13	(0.3)
COPD	13	(0.3)
Osteoporosis	10	(0.2)
Distant Metastasis	717	(13.9)
CCI score	0.75±2.18	

Abbreviations: CCI = Charlson Comorbidity Index; AIs = aromatase inhibitors.

### Incidence of fracture

**Fig 2:**
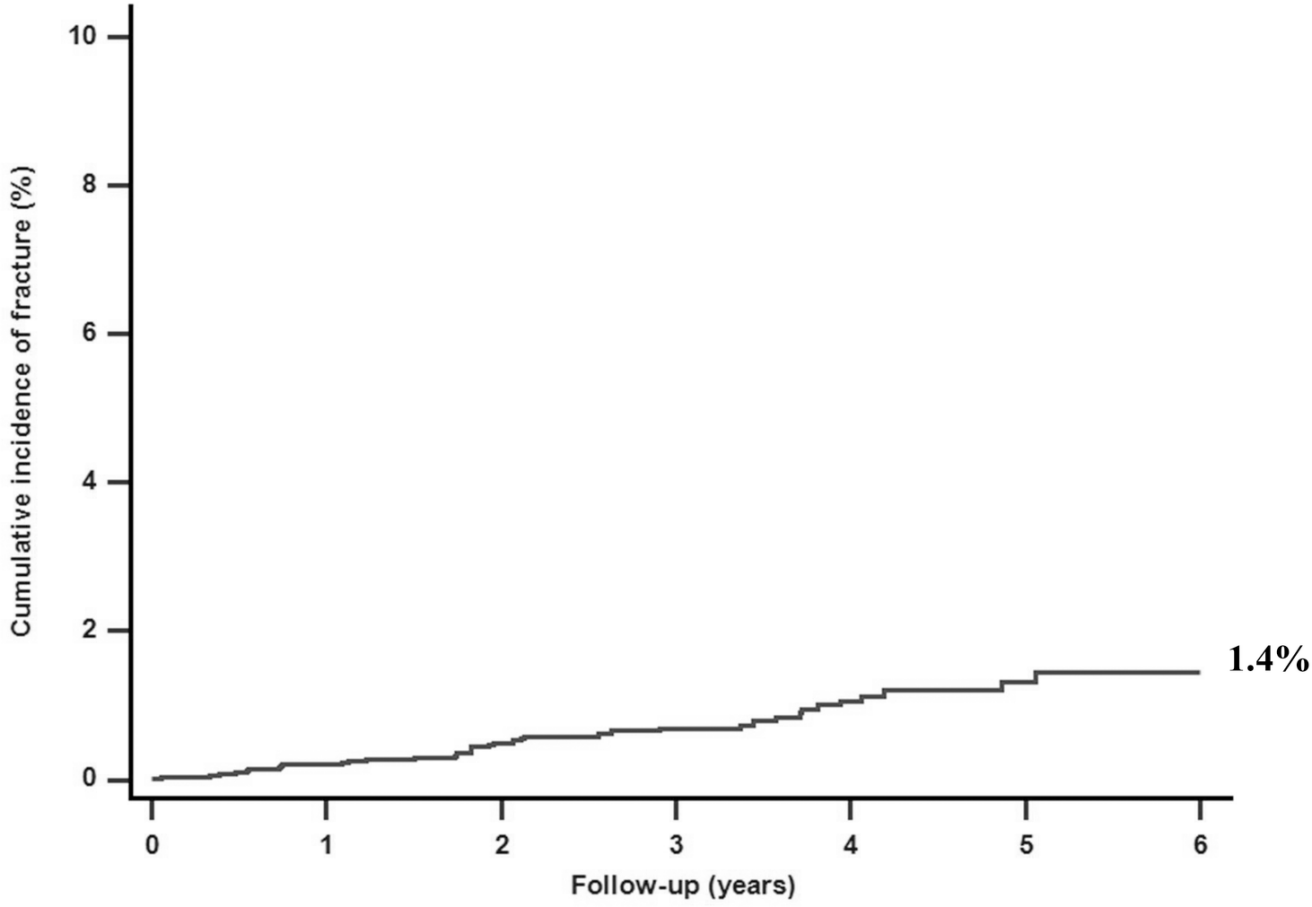
Cumulative incidence of fracture in young breast cancer cohort by Kaplan-Meier analysis (n = 5,146).

**Fig 3:**
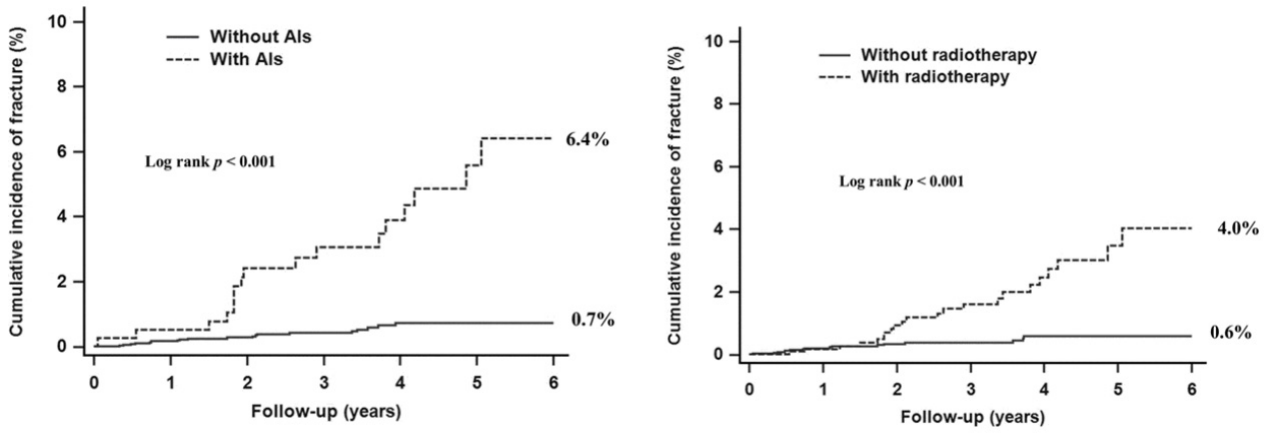
Cumulative incidence of fracture in young breast cancer patients according to the condition of (a) using or not using AIs, and (b) receiving or not receiving radiotherapy.

The incidence of subsequent fractures of breast cancer survivors was 54.34 per 10,000 person-years. The cumulative incidence of fracture in young breast cancer patients was 1.4% (Fig 2). Moreover, young patients who received AIs exhibited a significantly higher risk of fracture than that of those who did not (cumulative incidence 6.4% vs 0.7%, p < 0.001). Similarly, patients who received radiotherapy exhibited a higher risk of fracture than that of those who did not (cumulative incidence 4.0% vs 0.6%, p < 0.001) (Fig 3).

### Risk factors associated with fracture in breast cancer patients receiving adjuvant therapies

As shown in Table 2, we performed univariate analysis to predict the risk of fracture in young breast cancer patients; the results demonstrated that patients who received tamoxifen, AIs, trastuzumab, chemotherapy, and radiotherapy were at a higher risk. After age and comorbidities were controlled for, the Cox multivariate proportional hazards analysis showed that AIs (adjusted HR [aHR] = 7.221, 95% CI = 3.743–13.929, p < 0.001), trastuzumab (aHR = 5.032, 95% CI = 2.091–12.112, p < 0.001), and radiotherapy (aHR = 4.403, 95% CI = 2.271–8.536, p < 0.001) were significantly associated with a higher risk of fracture.

**Table 2:** Cox regression analysis of fracture incidence in young breast cancer survivors with adjuvant treatments.

Type of Adjuvant therapies	Univariate analysis			Multivariate analysis		
	HR	95% CI of HR	p-value	aHR	95% CI of aHR	p-value
Selective oestrogen receptor modulator	1.782	(0.814, 3.898)	0.148	1.825	(0.831, 4.008)	0.134
AIs	7.251	(3.786, 3.889)	< 0.001	7.221	(3.743, 13.929)	<0.001
Monoclonal antibody	4.899	(2.041, 11.759)	< 0.001	5.032	(2.091, 12.112)	<0.001
Chemotherapy	1.854	(0.943, 3.643)	0.073	1.856	(0.944, 3.650)	0.073
Radiotherapy	4.447	(2.307, 8.573)	<0.001	4.403	(2.271, 8.536)	<0.001

Abbreviations: AIs = aromatase inhibitors; HR = hazard ratio; aHR = adjusted hazard ratio.

Multivariable analysis including age, and comorbidities of hypertension, autoimmune diseases, DM, COPD, and osteoporosis.

As shown in Table 3, we focused on the incidence of fracture in patients receiving AIs and radiotherapy. The risk of fracture was high in patients receiving AIs. Compared with the patients who received AIs for fewer than 180 days, those who received AIs for more than 180 days had a high HR of 1.77 (95% CI = 0.68–4.57), which was not significant. However, the HR of fracture was 2.54 (95% CI = 1.07–6.06) in patients who received more than four radiotherapy visits. Moreover, we further investigated the risk of fracture at three sites and listed the observations in Table 4. Under the site-specific analysis, young breast cancer patients who received AIs had the highest risk of hip fracture (HR = 8.520, 95% CI = 1.711–42.432, p < 0.04), whereas patients who received radiotherapy had the highest risk of vertebral fracture (HR = 5.512, 95% CI = 1.847–16.451, p < 0.01) (Table 4).

**Table 3:** Risk of fracture in young breast cancer patients receiving AIs or radiotherapy.

	N	Events	Person-years	IR	HR(95% CI)	p-value^1^	p-value^2^
AIs prescription days							
≤180	204	6	722	8.31	1.00(Reference)	0.2553	0.2164
>180	191	11	774	14.21	1.77(0.68, 4.57)		
Radiotherapy visits							
≤4	825	9	2411	3.73	1.00(Reference)	0.0258*	0.0202*
>4	419	13	1344	9.67	2.54(1.07, 6.06)		

Abbreviations: AIs = aromatase inhibitors; HR, hazard ratio.

IR, incidence rate, per 1000 person-years.

^1^p-value: estimated using Cox regression models.

^2^p-value: exact two-tailed probability estimated using hypergeometric tests.

**Table 4:** Hazard ratio and 95% confidence intervals of site-specific fracture in young breast cancer group receiving AIs or radiotherapy.

	Hip fracture	Vertebral fracture	Limb fracture
	HR (95% CI)	HR (95% CI)	HR (95% CI)
AIs	8.520 (1.711, 42.432)*	8.212 (2.858, 23.597)***	7.116 (2.938, 17.240)***
Radiotherapy	3.001 (0.606, 14.869)	5.512 (1.847, 16.451)**	3.016 (1.255, 7.247) *

Abbreviations: AIs = aromatase inhibitors; HR, hazard ratio.

*p<0.05

**p<0.01

***p<0.001.

## Discussion

Based on our research, this is the first study to analyze the subsequent risk of fracture in young breast cancer survivors aged 20–39 years who received adjuvant therapies. The results of this nationwide population-based cohort study show that the incidence rate of fracture in young breast cancer survivors was 1.4%. The risk of fracture was higher in patients who received AIs or radiotherapy than in those who did not. A higher number of radiotherapy visits exposed patients to a higher risk of fracture. Patients who received AIs had the highest HR for hip fracture whereas patients who received radiotherapy had the highest HR for vertebral fracture.

A study using a population-based representative sample showed that the incidence of fracture in the general Taiwanese female population was approximately 42.52 per 10,000 [10]. In this study, 561 fractures were reported among 37,231 breast patients. The relative risk ratio (RR) of fracture in breast cancer patients was (95% CI = 3.185–3.947, p < 0.001). Therefore, the incidence of fracture in breast cancer patients was significantly higher than that in the general population [10]. Moreover, we compared the incidence of subsequent fractures between young breast cancer patients and young non breast cancer patients from the Taiwan Cancer Registry database [3]. The cumulative incidence of fracture in our study was 1.4% for the young breast cancer group, which is significantly higher than 0.3% for the young non breast cancer patients in Taiwan (RR = 4.762, 95% CI = 3.996–5.764, p < 0.001).

Furthermore, according to our research, our study is the first to report a high risk of fracture following adjuvant therapies including AIs, radiotherapy, and monoclonal antibodies. Young breast cancer patients who received AIs had a higher aHR than that of those who did not (aHR = 7.221, 95% CI = (3.743, 13.929), p < 0.001). An increase in the number of bony fractures has been reported in clinical trials on AIs used in treating breast cancer patients [24–26]. A population-based cohort of 2,003 older female breast cancer patients revealed that patients who received AIs had a higher risk of fracture (aHR = 1.34, CI = [0.92, 1.94]) [12]. In another study on 211 postmenopausal breast cancer patients, the aHR was 20.08 (95% CI = 1.72–234.08, p = 0.017) in patients who received endocrine therapy compared with those who did not (1.1%, 1/89) [13]. However, these two studies have focused on postmenopausal patients. Our study is the first to investigate the risk of fracture on receiving AIs in young patients. Furthermore, our results showed that radiotherapy and monoclonal antibodies were significantly associated with a high risk of fracture.

Furthermore, we evaluated the dose effect by using the prescription duration for AIs and the number of visits for radiotherapy because the radiation dose-related details were not available in the NHIRD, which is one of the limitations of this study. Few patients received monoclonal antibodies and developed fractures; therefore further analysis could not be performed because of the small sample size. Young breast cancer patients who received AIs for more than 180 days or those who received more than four radiotherapy visits had a higher risk of fracture (HR: 1.77 and 2.54, respectively, although the HR was not statistically significant for AIs). Additional studies are necessary to investigate the dose effects of AIs and radiotherapy.

In the site-specific analysis, limbs were most common fracture sites, and this finding is consistent with those of a review on adult fractures [27]. However, we observed that young patients who received AIs had the highest risk of hip fracture whereas young patients who received radiotherapy had the highest risk of vertebral fracture. An increased risk of vertebral fracture has been reported in previous studies. Kanis et al. reported that the incidence of vertebral fracture was nearly five times higher than normal in women from the time of the first diagnosis (odds ratio [OR] = 4.7, 95% CI = 2.3–9.9) [28], while Chen et al. reported that the increased risk of vertebral fracture was significant among breast cancer before the age of 55 years (HR = 1.78, 95% CI = 1.28–2.46) [9]. Another study showed that the incidence of fracture was higher in the breast cancer cohort than in the comparison cohort with aHR of 1.24 (95% CI = 1.04–1.48) for vertebral fractures [10]. However, the possible mechanisms of the association between breast cancer and vertebral fractures remain unknown.

To date, the pathophysiological mechanisms for fracture following treatment using AIs have not yet been clearly identified. Studies have revealed early breast cancer women who received AIs are at a risk of bone loss, which resulted from AI-induced nearly complete inhibition of estrogen production [29, 30]. AIs may cause estrogen suppression and increase bone turnover rates, thus accelerating bone loss. Therefore, these effects may ultimately result in osteoporosis and an increased risk of fracture, particularly at the hip [31–33].

The relationship between radiotherapy and fracture may be associated with the chest wall radiation therapy because radiation therapy has been commonly used to minimize the risk of local cancer recurrence after lumpectomy [2, 8]. Frequent chest wall radiation may increase the finding of vertebral fracture. Bone metastases are the most common manifestation of metastatic breast cancer and are associated with high morbidity [34]. Therefore, vertebral metastasis may result in a high risk of vertebral fracture.

Our study has some limitations. First, the NHIRD did not provide information on potential confounders such as cancer staging, pregnancy or lactation status, bone mineral density, lifestyle habits (smoking, alcoholism, and so on), the use of self-paid medications (vitamin D, calcium, and so on). However, young breast cancer patients are less affected by risk factors such as age or menopause compared with older patients [2]. Adjuvant therapies affect younger patients more significantly than older patients [21]. Therefore, our study results may be heavily influenced by adjuvant therapies. Second, the fractures may have been diagnosed before 2000, and these data could not be detected or excluded in this study. Third, patients with minor fractures may not seek treatment or be aware of their fractures. However, these data were distributed equally between two groups (those who received adjuvant therapies and those who did not) and caused no bias in the results.

In conclusion, this study found a significant subsequent risk of fracture following adjuvant therapies including AIs, radiotherapy, and monoclonal antibodies. Furthermore, these risks may increase with the duration of AI use and radiotherapy. Patients who received AIs had the highest risk of hip fractures, whereas those who received radiotherapy had the highest risk of vertebral fractures. Therefore, young breast cancer patients who are receiving adjuvant therapies such as AIs, radiotherapy, or monoclonal antibodies need to be more careful for preventing fracture events. Breast cancer treatment plans are suggested to incorporate fracture prevention interventions.
